# Optimization of Two Steps in Scale-Up Synthesis of Nannocystin A

**DOI:** 10.3390/md19040198

**Published:** 2021-03-31

**Authors:** Tingrong Zhang, Shaojie Miao, Mingxiao Zhang, Wenjie Liu, Liang Wang, Yue Chen

**Affiliations:** State Key Laboratory of Medicinal Chemical Biology, College of Pharmacy, and Tianjin Key Laboratory of Molecular Drug Research, Nankai University, Tianjin 300350, China; tingrongzhang@mail.nankai.edu.cn (T.Z.); miaoshaojie@mail.nankai.edu.cn (S.M.); mingxiaozhang@mail.nankai.edu.cn (M.Z.); liuwenjie@mail.nankai.edu.cn (W.L.)

**Keywords:** total synthesis, natural product, nannocystin, anti-cancer, gram-scale

## Abstract

We have accomplished a 10-step (longest linear) total synthesis of nannocystin A on a four hundred milligram scale. The previously reported Kobayashi vinylogous Mukaiyama aldol reaction to connect C4 and C5 was unreproducible during the scaling up process. A more convenient and cost-efficient Keck asymmetric vinylogous aldol reaction was employed to improve this transformation.

## 1. Introduction

Marine myxobacteria are prolific producers of secondary metabolites owning unique structures and exhibiting multiple biological activities ranging from antibiotic to anti-cancer [1,2]. The discovery of epothilones [3] from myxobacteria and their metabolic stable analogue Ixabepilone [4,5] (approved for the treatment of aggressive breast cancer) for clinical use highlight the powerful potential of myxobacteria as resources for drug discovery. More importantly, novel modes of action [6] were also identified during the pharmacological study of these myxobacteria-derived natural products, and hereby proceeded the target-oriented drug discovery. 

**Figure 1:**
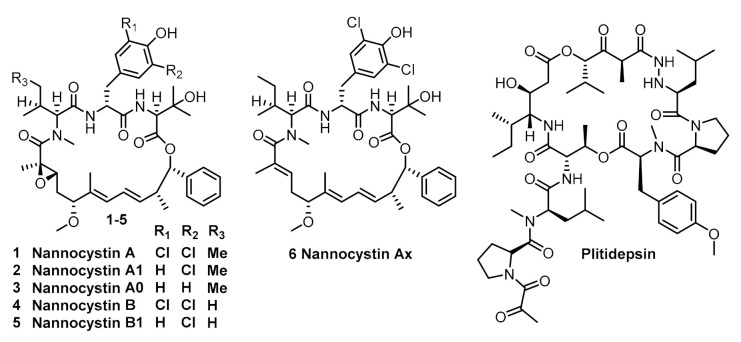
Structure of nannocystins and plitidepsin.

Nannocystin A (**1**) and its natural congener (**2**–**6**) (Figure 1) are myxobacterial secondary metabolites isolated by Hoepfner [7] and BrÖnstrup [8] independently from *Nannocystis sp*. They exhibit significant inhibitory activity against a broad variety of human cancer cells at nanomolar concentrations [7,8]. This anti-neoplastic activity is attributed to the binding affinity with elongation factor 1A (eEF1A) [7]. Since this mechanism is shared by plitidepsin [9,10] (isolated from the marine tunicate *Aplidium albicans*, Figure 1) which has recently been approved by the Australia Therapeutic Goods Administration for clinical use against multiple myeloma, it is obvious that nannocystins might be a promising lead for anti-cancer drug discovery.

Structurally, nannocystins share a rigid 21-membered macroskeleton bearing nine chiral centers (7 chiral centers for **6**), an N-methyl α,β-epoxy amide (for **1**–**5**) and two conjugated E-alkenes. Its novel macroskeleton, strong antineoplastic activity and unusual mechanism attracted the interests of the chemical community. Thus far, seven total syntheses [11,12,13,14,15,16,17,18,19,20] of **1** and **6** have been finished. For efficiency, all synthetic routes fully utilized the principle of convergency, endowing freedom of structural modification of individual moieties, which help to uncover the preliminary of structure-activity relationship of the macroskeleton [17,21,22,23,24]. However, the quantities of nannocystins obtained from previously reported studies are insufficient for multipronged biological testing. Only Liu and Ye reported 75 mg and 20 mg-scaled synthesis of nannocystins, respectively, whereas others (including us) reported it on a milligram scale. This might be an explanation that the biological testing of nannocystin A is stagnant at the in vitro level, and tardily cannot advance to the in vivo level. To address the supply issue for in vivo study, we enlarged the synthetic procedures we previously reported. Herein, we describe the details of our efforts in the optimization of some procedures. The key improvement is that a Keck asymmetric vinylogous Mukaiyanma aldol reaction was employed to construct the carbon bond between C4 and C5. We finally obtained 420 mg of nannocystin A for future biological testing.

## 2. Results

### Chemistry

**Figure 2:**
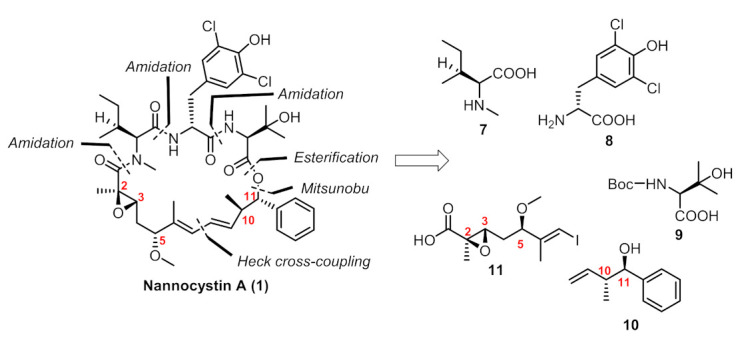
Retrosynthetic analysis of our route.

**Figure 3:**
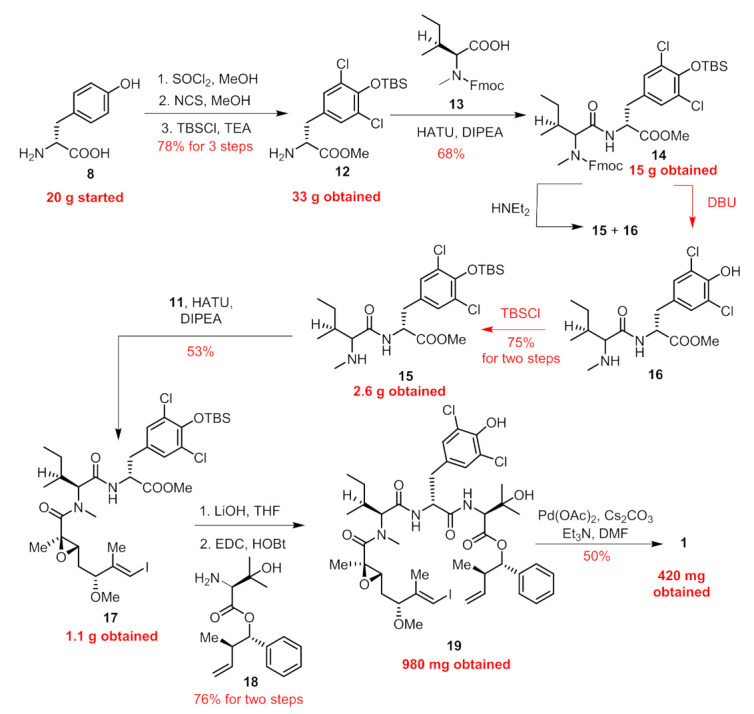
Previously reported synthetic route towards **1**, scaled up synthesis for the current batch was marked red.

In our previous paper [13], we provided a concise route in 10 steps (longest linear sequence) featuring an intramolecular Heck cross-coupling for the final macrocyclization. Connections of building blocks (**7**–**11**, Figure 2) via well-established esterification, amidation and the Mitsunobu reaction succeeded in providing the penultimate linear precursor **19** (Figure 3). Considering that five chiral centers (C2, C3, C5, C10, C11) were built on our own and the other four were innate in commercially available amino acid-derived starting materials, we paid key attention to these five chiral centers during the amplification process. 

We first amplified the synthesis of **18** (structure shown in Figure 3). The Mitsunobu reaction between anti-homoallylic alcohol **10** and N-Boc-3-hydroxy-D-valine **9** went smoothly according to a previously reported procedure to give **18** in 70% yield, with 12 g obtained for the current batch.

**Figure 4:**
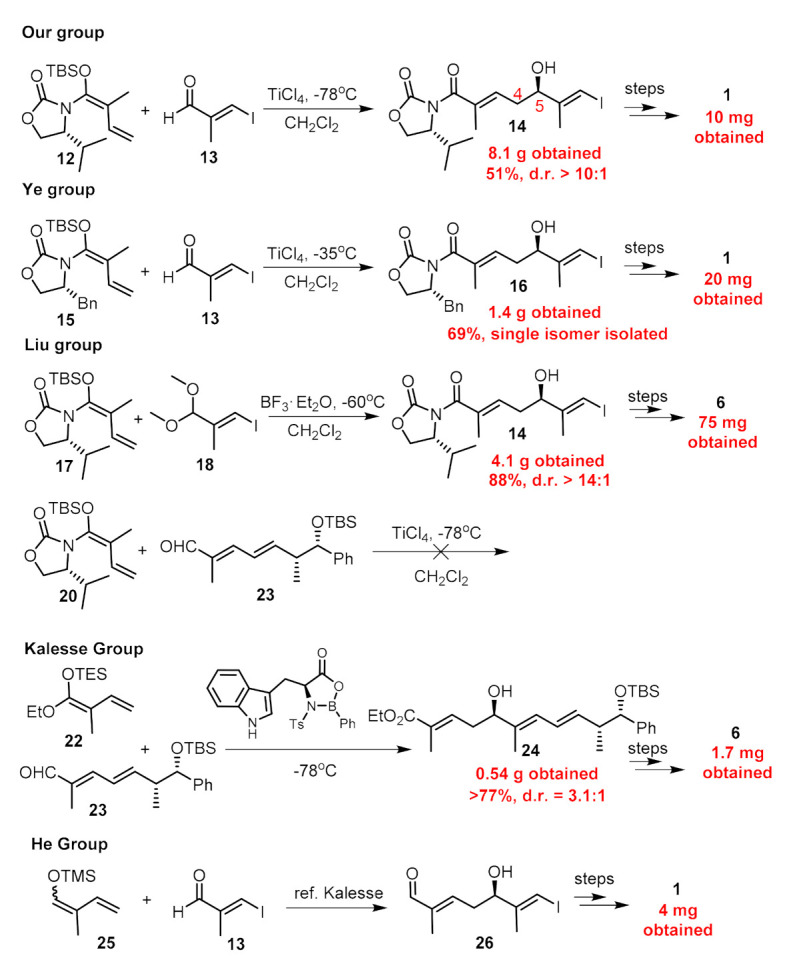
The methods of C4–C5 carbon bond construction using Mukaiyama aldol reaction and the quantities of the natural products previously obtained by other researchers.

As for the establishment of the C5 chiral center, all seven reported synthetic routes deployed nucleophilic attack of carbon anions towards carbonyl groups, five of which including us employed an asymmetric vinylous Mukaiyama-type aldol reaction (Figure 4). Kobayashi et al. [25] first developed this type aldol reaction of an aldehyde with vinylketene silyl N,O-acetal, and the enantio-selectivity was controlled by the remote Evans auxiliary. By employing this methodology, we produced **14** (Figure 4) in an acceptable yield with a d.r. value > 10:1, and 8.1 g of product **14** was obtained for the previous batch. However, when we reperformed this reaction, we found it was capricious because upon scale-up to 5 g, the yield dropped considerably to 10%. Strictly following the previous operation, we repeated this reaction several times; the yield can occasionally reach up to 50% but it was unreproducible. In most cases, the yield ranged from 5% to 20%. Then, we carefully checked the details of this reaction such as the purity of reactants and solvent, the equivalents and concentrations of reactants, the reaction time and temperature, the stirring speed, the method of quench, etc., but still failed in furnishing **14** in a stable yield more than 20%. Meanwhile, Liu et al. [20] also reported the fruitlessness during the synthesis of nannocystin Ax utilizing **20** and aldehyde **23** (Figure 4). They found both reactants decomposed rapidly under treatment with Lewis acid such as TiCl_4_. In addition, considering the potential hazards of TiCl_4_ in the amplification process, we set out for an alternative method to achieve this transformation. 

**Figure 5:**
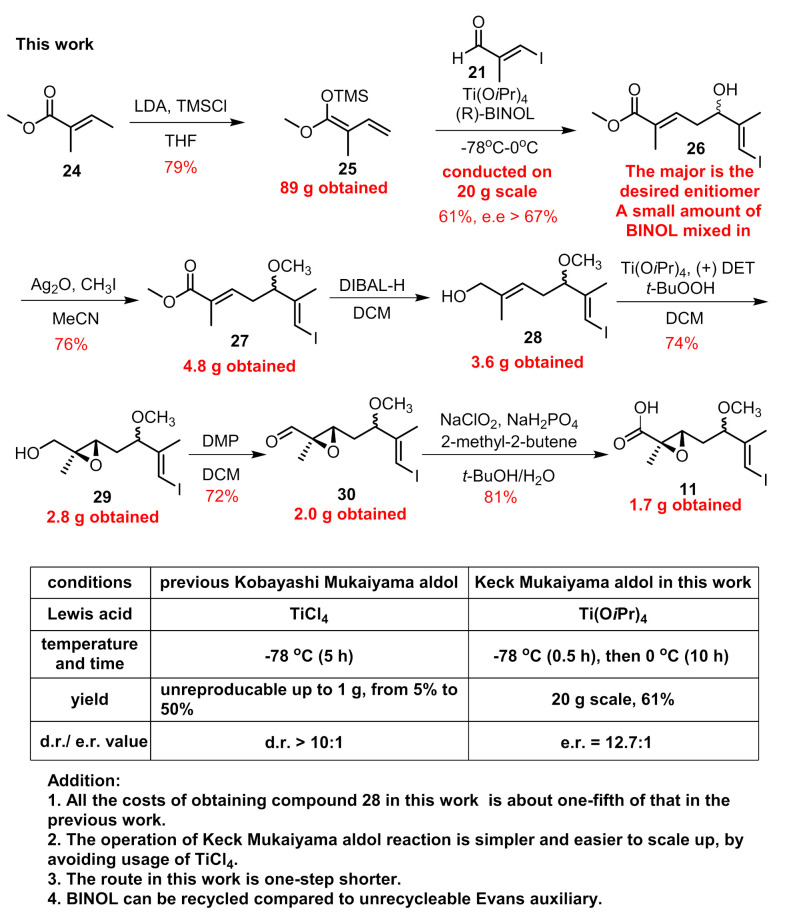
The optimization of vinylous Mukaiyama aldol reaction.

Ti(O*i*Pr)_4_ is a mild reagent compared to TiCl_4_, and its combination with BINOL can also mediate the asymmetric Mukaiyama-type aldol reaction between aldehyde and vinylketene silyl acetal [26]. By employing the chiral BINOL reagents, this reaction can proceed in an enantio-selective manner with high e.e. value evidenced by our recent synthetic work of ovatodiolide [27]. Besides, the external addition of BINOL can save the installation and removal of auxiliaries on reactants compared to Evans auxiliary methodology, and BINOL can be recovered after the reaction is completed. Then, we chose economical material **24** (Figure 5) to form **25** as the coupling partner on a milligram scale. The reaction between aldehyde **21** and **25** with the addition of Ti(O*i*Pr)_4_ and (*R*)-BINOL went smoothly to provide **26** in a stable >55% yield with an e.e. value = 85% [13]. The temperature was maintained at −78 °C only for 30 min after the reaction began and was allowed to warm to 0^o^C for another 10 h stirring. By far, the biggest batch we preformed was 20 g for compound **21** without any erosion of yield (for a complete comparison between two vinylous Mukaiyama aldol reactions, see Figure 5). Since the two produced enantiomers could not be easily separated, purification was deferred to later steps. Compound **26** was then converted to **27** under treatment with Ag_2_O and CH_3_I. The reduction of the methyl ester group with DIBAL-H afforded us 3.6 g of allylic alcohol **28**. With the aid of Sharpless’ conditions, epoxidation proceeded stereoselectively and we obtained **29** as a mixture of two diastereomers. Next, building block **11** was obtained via two successive oxidations on a gram scale according to previous procedures. The compound with an undesired configuration at C5 disappeared after condensation with amine **15** according to the ^1^H-NMR of isolated product **17**.

To shorten steps, we also attempted direct epoxidation using vinyl ester **27** to give **31** (see the Supporting Information) [28,29,30], which could simply hydrolyze to provide building block **11.** However, after testing several conditions, we found this transformation was unsuccessful, with only a trace amount of the desired product obtained. Thus, we gave it up and turned our emphasis to the amplification of other moieties.

As shown in Figure 3, following previous procedures, 15 g of compound **14** was obtained unimpededly. However, the removal of the Fmoc group with Et_2_NH was problematic when scaled up to 1 g. The *t*-butyldimethylsilyl (TBS) group could be simultaneously cleaved partially. We extended the reaction time and increased the equiv. of Et_2_NH, but still TBS could not be cleaved entirely and two products (**15** and **16**) were detectable through thin-layer chromatography (TLC) analysis. The isolation process was cumbersome because the secondary amine was hard to remove. Therefore, we employed circuitous tactics. First, the treatment of **14** with 1,5-diazabicyclo[4.3.0]non-5-ene (DBU) rapidly delivered us **16**. With the concern that the phenol might make an impact on the following coupling, we unmasked it with a TBS group again to obtain **15**.

The subsequent transformation from **15** to the penultimate linear precursor proceeded smoothly to give rise to 980 mg of **19** for the current batch. By subjecting **19** to the intramolecular Heck macrocyclization, we finally obtained 420 mg of **1** in 50% yield (brsm). It was noteworthy that no cis/trans isomers were detected during this transformation.

## 3. Materials and Methods

### 3.1. General Information

Reagents were purchased from commercial suppliers and used without purification unless otherwise stated: lithium diisopropylamide (LDA), 1-(3-dimethylaminopropyl)-3-ethylcarbodiimide (EDC), N-chlorosuccinimide (NCS), Nhydroxybenzotrizole (HOBt), 1-(2-hydroxynaphthalen-1-yl)naphthalen-2-ol (BINOL), dichloromethane (DCM), Dess-Martin periodinane (DMP), 1-(bis(dimethylamino)methylene)-1H-1,2,3-triazolo(4,5-b)pyridinium 3-oxid hexafluorophosphate (HATU), and N,N-Diisopropy-lethylamine (DIPEA), *t*-butyldimethylchlorosilane (TBSCl), diisobutyl aluminium hydride (DIBAL-H).

All reactions were carried out under an argon atmosphere with dry solvents under anhydrous conditions, unless otherwise noted. Tetrahydrofuran (THF) was distilled immediately before use from sodium-benzophenone ketyl. Solvents for chromatography were used as supplied by Tianjin Reagents Chemical (Tianjin, China). Reactions were monitored by thin-layer chromatography (TLC) carried out on silica gel plates, using UV light as the visualizing agent and aqueous phosphomolybdic acid or basic aqueous potassium permanganate as the developing agent. A 200–300 mesh silica gel was used for column chromatography. 

Optical rotations were recorded on an Insmark IP 120 digital polarimeter (Insmark, Shanghai, China). IR spectra were recorded on a Bruker Tensor 27 instrument (Ettlingen, Germany). Only the strongest and/or most structurally important absorptions of IR spectra were reported in wavenumbers (cm^−1^). ^1^H NMR, ^13^C NMR, and 2D NMR were recorded on Bruker AV 400 and calibrated by using internal references and solvent signals CDCl_3_ (δ_H_ = 7.26 ppm, δ_C_ = 77.16 ppm) and CD_3_OD (δ_H_ =3.31 ppm, δ_C_ = 49.0 ppm), unless otherwise noted. ^1^H NMR data are reported as follows: chemical shift, multiplicity (s = singlet, d = doublet, t = triplet, q = quartet, p = quintet, br = broad, m = multiplet), coupling constants and integration. High-resolution mass spectra (HRMS) were detected on an IonSpec Fourier transform ion cyclotron resonance mass spectrometer by Varian 7.0T FTMS (Kuala Lumpur, Malaysia).

### 3.2. Chemistry

Compounds **8**, **12**, **17**, **18**, **19**, **1**, **29**, **30**, and **11** were obtained following the procedure reported previously.

*Methyl-(2R)-3-(3,5-dichloro-4-hydroxyphenyl)-2-((3S)-3-methyl-2-(methylamino)pentanamido) propanoate (**16**)*

To a solution of compound **14** (5.0 g, 6.9 mmol) in dry DCM (100 mL), DBU (5 mL, 33.4 mmol) was added. The reaction mixture was stirred for 30 min at room temperature. The reaction was quenched by silica gel, then purified by column chromatography (DCM:MeOH=50:1) to give the product **16** (2.7 g, crude) as a colorless oil.

*Methyl-(2R)-3-(4-((tert-butyldimethylsilyl)oxy)-3,5-dichlorophenyl)-2-((3S)-3-methyl-2-(methylamino)pentanamido)propanoate (**15**)*

To a solution of **16** (2.7 g, 6.9 mmol) in dry DCM (30 mL), triethylamine (1.9 mL, 13.8 mmol) was added, followed by TBSCl (1.6 g, 10.4 mmol) under ice bath. The reaction was stirred for 2 h at room temperature. Then, the reaction was quenched by water (10 mL), extracted with DCM (15 mL × 3). The combined organic layers were dried over MgSO_4_, filtered and concentrated to give the crude. The crude was purified by column chromatography (DCM:MeOH, 100:1–70:1) to give the product **15** (2.6 g, 75% for two steps) as a colorless oil. The spectroscopic data are consistent with those reported in the literature.

*(Z)-((1-methoxy-2-methylbuta-1,3-dien-1-yl)oxy)trimethylsilane (**25**)*

To a solution of diisopropylamine (67 g, 0.66 mol) in dry THF (250 mL), *n-*BuLi (266 mL, 2.5 M in hexane) was added at −78 °C. The reaction mixture was warmed to 0 °C for 30 min, then cooled to −65 °C. Methyl tiglate **24** (68 g, 0.60 mol) in THF (30 mL) was added dropwise. The reaction mixture was stirred for 2 h, and after that, TMSCl (78 g, 0.72 mol) in THF (30 mL) was added dropwise. Then, the reaction was warmed to room temperature at a period of 4 h, diluted by hexane (1000 mL), and then filtered and concentrated to give a crude. The crude was distilled at 80 °C under reduced pressure to give the compound **25** as a light yellow liquid (89 g, 79%), which was directly used in the next step. 

*Methyl-(2E,6E)-5-hydroxy-7-iodo-2,6-dimethylhepta-2,6-dienoate (**26**)*

To a solution of *R*-BINOL (15.2 g, 53 mmol) and CaH_2_ (2.2 g) in dry THF (200 mL), Ti(O*i*Pr)_4_ (15.1 g, 53 mmol) was added at room temperature. The mixture turned orange while adding it. Then, the mixture was cooled to −78 °C after stirring at room temperature for 30 min, and then aldehyde **21** (21 g, 107 mmol) in THF (20 mL) was added dropwise, followed by dropwise addition of a solution of **25** (20 g, 107 mmol) in THF (20 mL). The mixture was then stirred at −78 °C for 30 min, and warmed to 0 °C for 10 h of stirring. Then, the reaction was quenched by saturated aqueous NaHCO_3_ (20 mL) and Rochelle salt (20 mL), extracted with EtOAc (80 mL × 3). The combined organic layers were dried over Na_2_SO_4_, filtered, and concentrated to give a crude. The crude was purified by column chromatography (PE:EA = 10:1), then redissolved by hexane, filtered, and concentrated to give the product **26** (20 g, 61%) as a yellow oil. ^1^H NMR (400 MHz, CDCl_3_) δ 6.72 (td, *J* = 7.2, 1.7 Hz, 1H), 6.31 (s, 1H), 4.29 (t, *J* = 6.5 Hz, 1H), 3.72 (s, 3H), 3.57 (d, *J* = 29.5 Hz, 1H), 2.44 (t, *J* = 6.9 Hz, 2H), 1.83 (d, *J* = 2.4 Hz, 6H). ^13^C NMR (101 MHz, CDCl_3_) δ 168.55, 149.18, 137.84, 129.46, 78.94, 75.11, 51.97, 34.55, 20.03, 12.72. IR(KBr)ν_max_: 3445, 2936, 1699, 1437, 1275, 1084, 795, 661 cm^−1^ [M + Na] calculated 332.9964 found 332.9969.

*Methyl-(2E,6E)-7-iodo-5-methoxy-2,6-dimethylhepta-2,6-dienoate (**27**)*

To a solution of **26** (20 g, 64.5 mmol) in dry MeCN (120 mL), Ag_2_O (37 g, 161 mmol) was added at room temperature, followed by methyl iodide (91 g, 645 mmol). Then, the reaction was stirred for 12 h at room temperature in a dark place. The reaction mixture was filtered through celite and concentrated to give a crude. The crude was purified by column chromatography (PE:EA = 20:1) to give methyl ether **27** (16 g, 49 mmol, 76%) as a yellow oil. ^1^H NMR (400 MHz, CDCl_3_) δ 6.69 (td, *J* = 7.2, 1.5 Hz, 1H), 6.27 (dd, *J* = 1.9, 1.0 Hz, 1H), 3.75 (dd, *J* = 7.6, 5.9 Hz, 1H), 3.74 (s, 3H), 3.21 (s, 3H), 2.53–2.42 (m, 1H), 2.41–2.31 (m, 1H), 1.83 (d, *J* = 1.4 Hz, 3H), 1.77 (d, *J* = 1.2 Hz, 3H). ^13^C NMR (101 MHz, CDCl_3_) δ 168.32, 147.10, 137.35, 129.45, 84.82, 79.73, 56.59, 51.82, 33.37, 29.71, 18.83, 12.70. IR(KBr)ν_max_: 2949, 1715, 1435, 1273, 1099, 797, 744, 660 cm^−1^ [M + Na] calculated 347.0120 found 347.0124.

*(2E,6E)-7-iodo-5-methoxy-2,6-dimethylhepta-2,6-dien-1-ol (**28**)*

To a solution of methyl ether **27** (4.8 g, 14.8 mmol) in dry DCM (40 mL), DIBAL-H (20 mL, 1 M in DCM) was added at −20 °C dropwise. After stirring for 30 min, the reaction was quenched by water (0.8 mL) and 15% aqueous NaOH (0.8 mL). The temperature was allowed to warm to room temperature. After that, water (2 mL) and MgSO_4_ (10 g) were added to the mixture, and it was stirred for 30 min and filtered through celite to give a crude. The crude was purified by column chromatography (PE:EA = 6:1) to give the product **28** (3.6 g, 82%) as a yellow oil. ^1^H NMR (400 MHz, CDCl_3_) δ 6.19 (s, 1H), 5.39–5.27 (m, 1H), 3.98 (s, 2H), 3.73–3.60 (m, 1H), 3.20 (d, *J* = 1.5 Hz, 3H), 2.36 (dt, *J* = 14.5, 7.1 Hz, 1H), 2.24 (dt, *J* = 14.6, 6.9 Hz, 1H), 1.94 (s, 1H), 1.76 (d, *J* = 1.4 Hz, 3H), 1.65 (s, 3H). ^13^C NMR (101 MHz, CDCl_3_) δ 147.49, 137.18, 120.77, 85.77, 79.20, 68.52, 56.46, 32.10, 18.83, 13.94. IR(KBr)ν_max_: 3675, 2950, 1473, 1261, 1094, 1030, 801 cm^−1^ [M + Na] calculated 319.0171 found 319.0170.

## 4. Conclusions

In summary, we have achieved a 10-step (longest linear) total synthesis of nannocystin A on a four hundred milligram scale. Two steps were found problematic when scaled up, especially for the difficulty we met when we scaled up with the Kobayashi Mukaiyama aldol reaction to construct the carbon bond between C4 and C5. In order to overcome it, we employed a more convenient and cost-efficient Keck asymmetric vinylogous aldol reaction, and we finally obtained four hundred milligrams of nannocystin A. By starting from the synthesis of nannocystin A on a large scale, it should be possible, at least at the outset, to scale the production of any synthetic nannocystin analogue for further lead optimization and preclinical development.

## Data Availability

Not applicable.

## Supplementary Materials

The following are available online at https://www.mdpi.com/article/10.3390/md19040198/s1, S1: conditions of direct epoxidation of compound **27**, S2: ^1^H and^13^C NMR spectra for synthesized new compounds. S3. The e.e. value of Mukaiyama aldol reaction is > 66% shown in ^1^H NMR of compound **29** and chiral HPLC data of compound **26**. S4. The determination of the absolute configuration by Mosher esters of compound **26.** S5. ^1^H NMR for compound **17**; ^1^H NMR and ^13^C NMR for nannocystin A.
