# Supplementary material for: Optimization of Two Steps in Scale-Up Synthesis of Nannocystin A
Source: Mar Drugs. 2021 Mar 31;19(4):198. doi: 10.3390/md19040198 (PMC8066987; doi:10.3390/md19040198)
Supplement: Supplementary file 1 [file marinedrugs-19-00198-s001.pdf]

Supporting Information

Optimization of Two Steps in Scale-up Synthesis of Nannocystin A

Tingrong Zhang, Shaojie Miao, Mingxiao Zhang , Wenjie Liu, Liang Wang* and Yue Chen*

S1. Conditions of direct epoxidation of compound 27

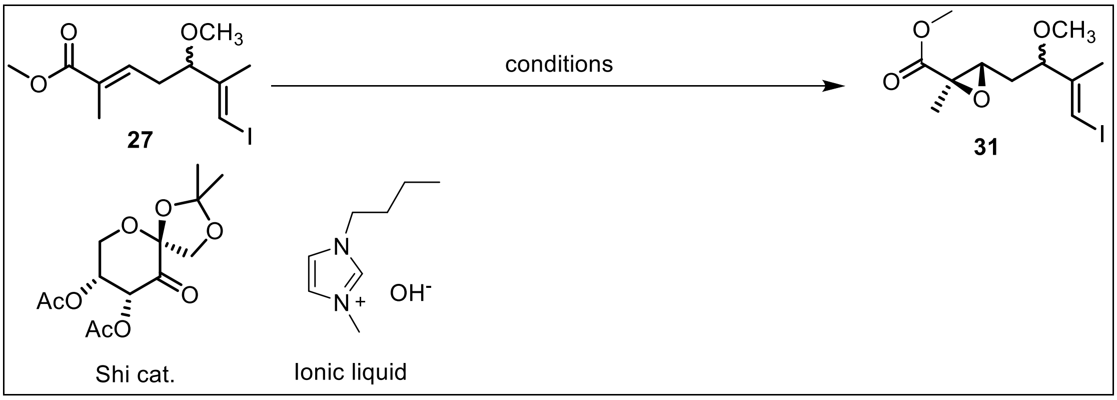

All the entries were excuted at room temperature unless extra notes.

entry	oxygen source	equivalent	Addition and equivalent	result
1	*m*-CPBA	1.5	DCM (25 V eq)[a]	messy
2	Oxone	1.1	DCM (25 V eq)	no reaction
3	*t*-BuOOH[b]	1.2	*S*-BINOL (0.08 eq), TPPO (0.1 eq), 4A MS (5 m), Y(O*i*Pr)$_3$ (0.08 eq), MeCN (100 V eq)	no reaction
4	*t*-BuOOH[b]	1.2	*S*-BINOL (0.08 eq), TPPO (0.1 eq), 4A MS (5 m), Y(O*i*Pr)$_3$ (0.08 eq), THF(100 V eq)	no reaction
5	Oxone	1	Shi cat. (0.15 eq), buffer (pH=7.4, 2 V), Na_2EDTA (2 eq), MeCN (5 V eq)	no reaction
6	Oxone	1	Shi cat. (0.15 eq), ionic acid (2 V eq), MeCN (1.5 V eq)	no reaction
7	H_2O_2 (30% in H_2O)	1.1	Shi cat. (0.15 eq), ionic acid (1.5V eq), MeCN(1.5V eq)	trace
8	Oxone	3	Shi cat. (0.15 eq), ionic acid (2 V eq), MeCN (1.5 V eq)	no reaction
9	H_2O_2 (30% in H_2O)	3.3	Shi cat. (0.15 eq), ionic acid (1.5 V eq), MeCN (1.5 V eq)	trace

a Excuted under ice bath.

b *t*-BuOOH was prepared from 40% solution in water and excessive Na_2SO_4.

S2. NMR Spectra of new compounds

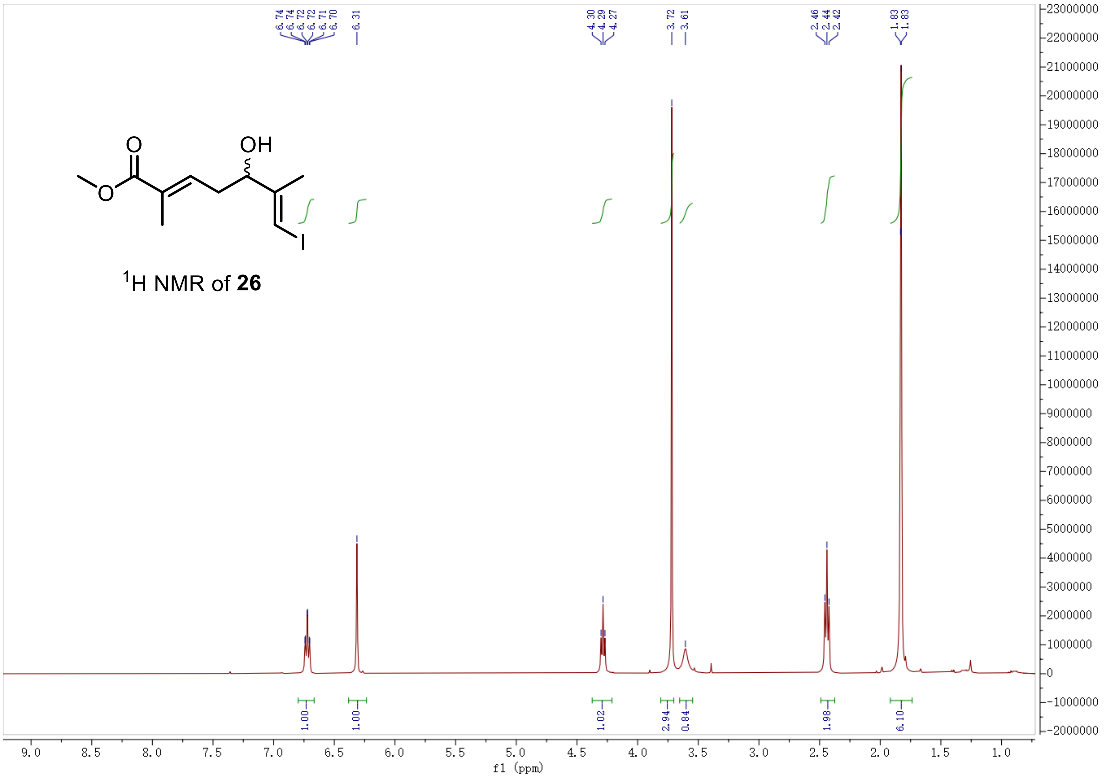

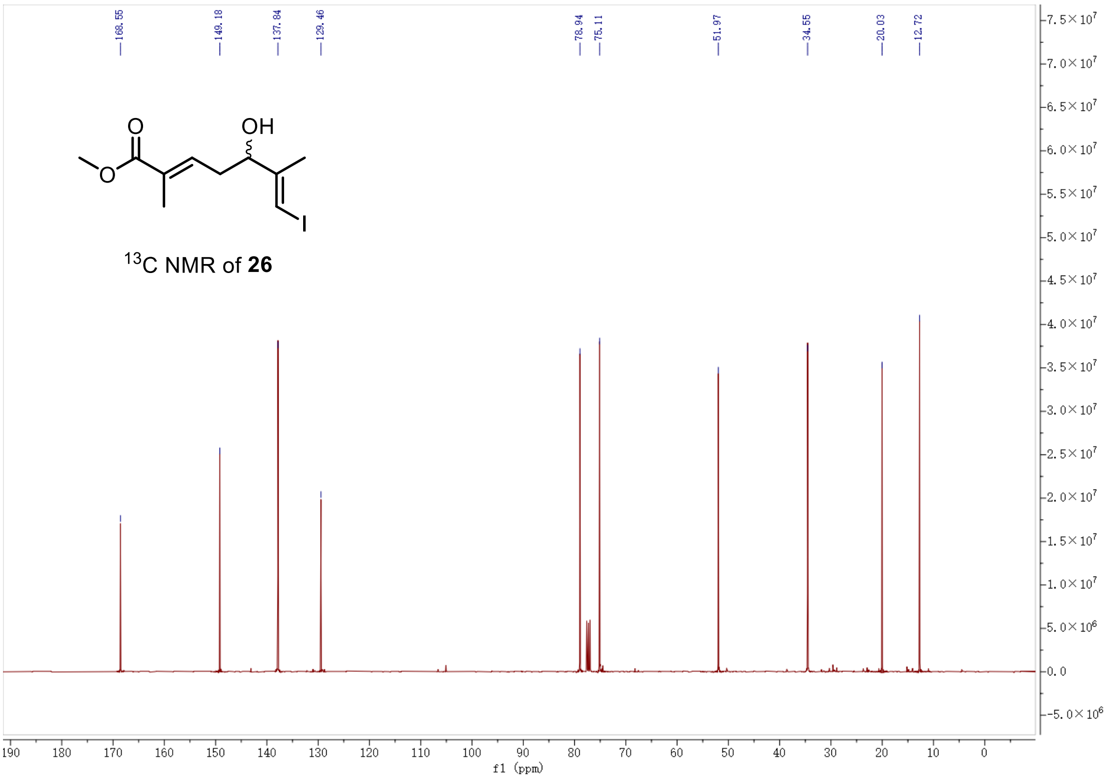

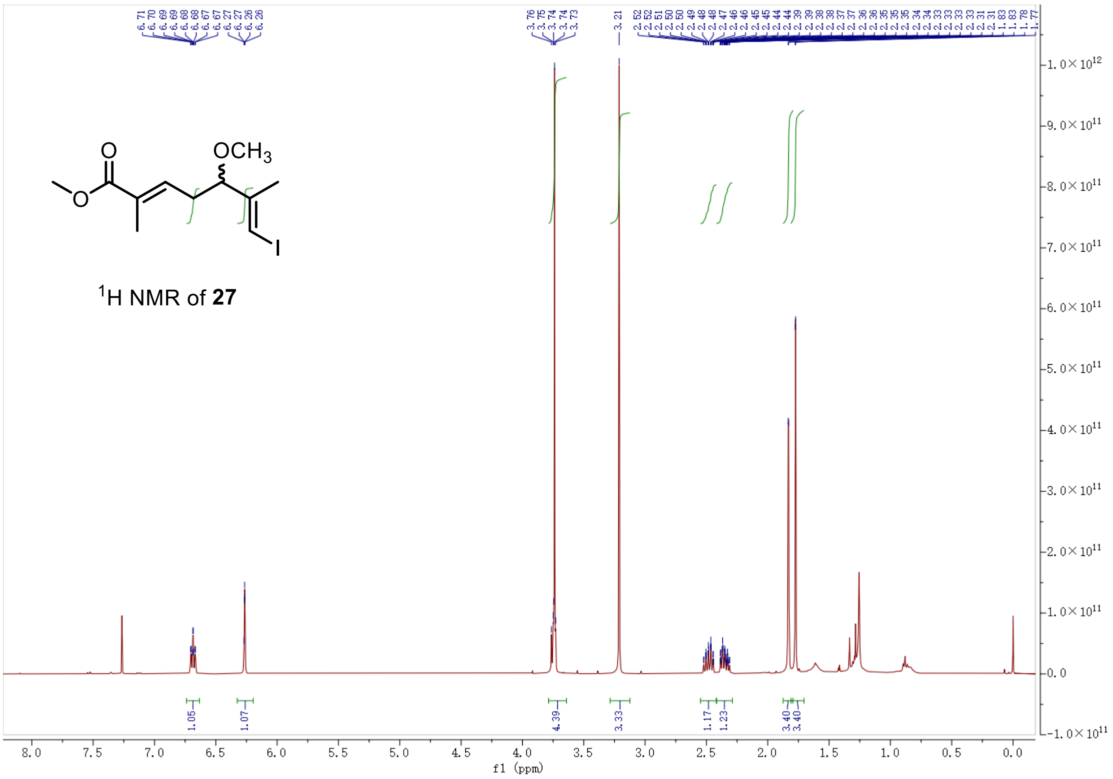
OCH3
I
1H NMR of 27
6.71 6.70 6.69 6.69 6.68 6.68 6.67 6.67 6.27 6.27 6.26 6.26
3.76 3.75 3.74 3.74 3.73
3.21
1.83 1.78 1.77
1.05
1.07
4.39
3.33
1.17
1.23
3.40
3.40
f1 (ppm)

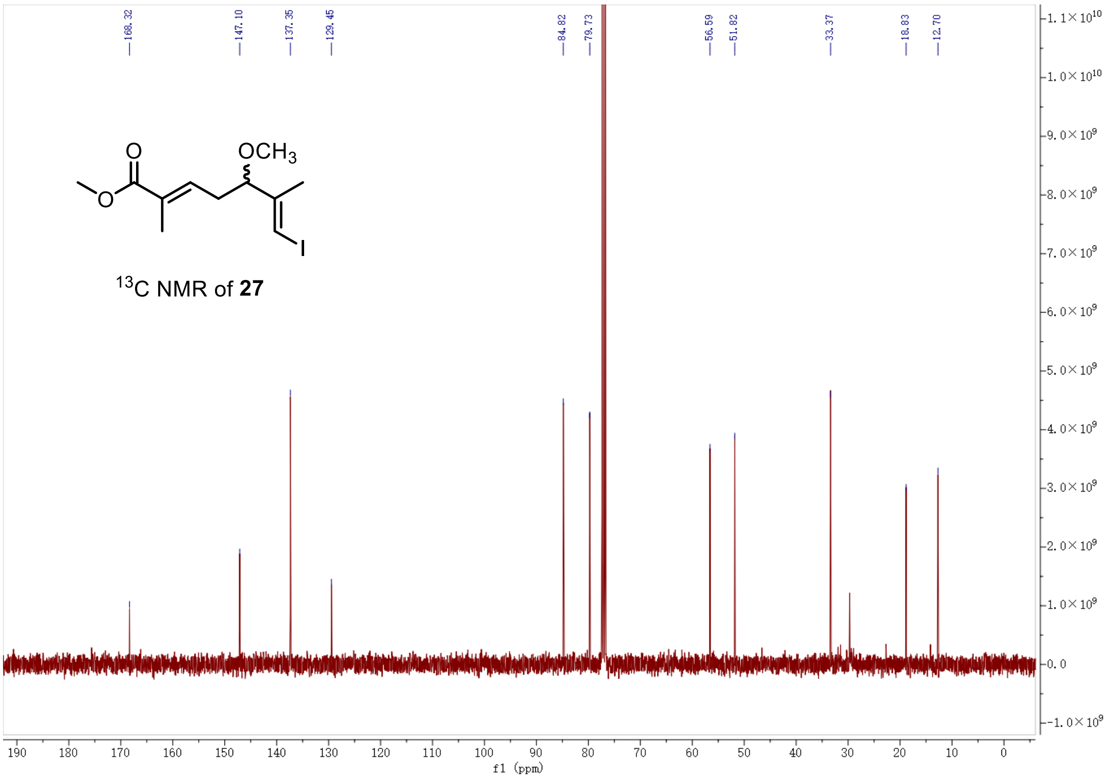
OCH3
I
13C NMR of 27
168.32
147.10
137.35
129.45
84.82
79.73
56.59
51.82
33.37
18.83
12.70
f1 (ppm)

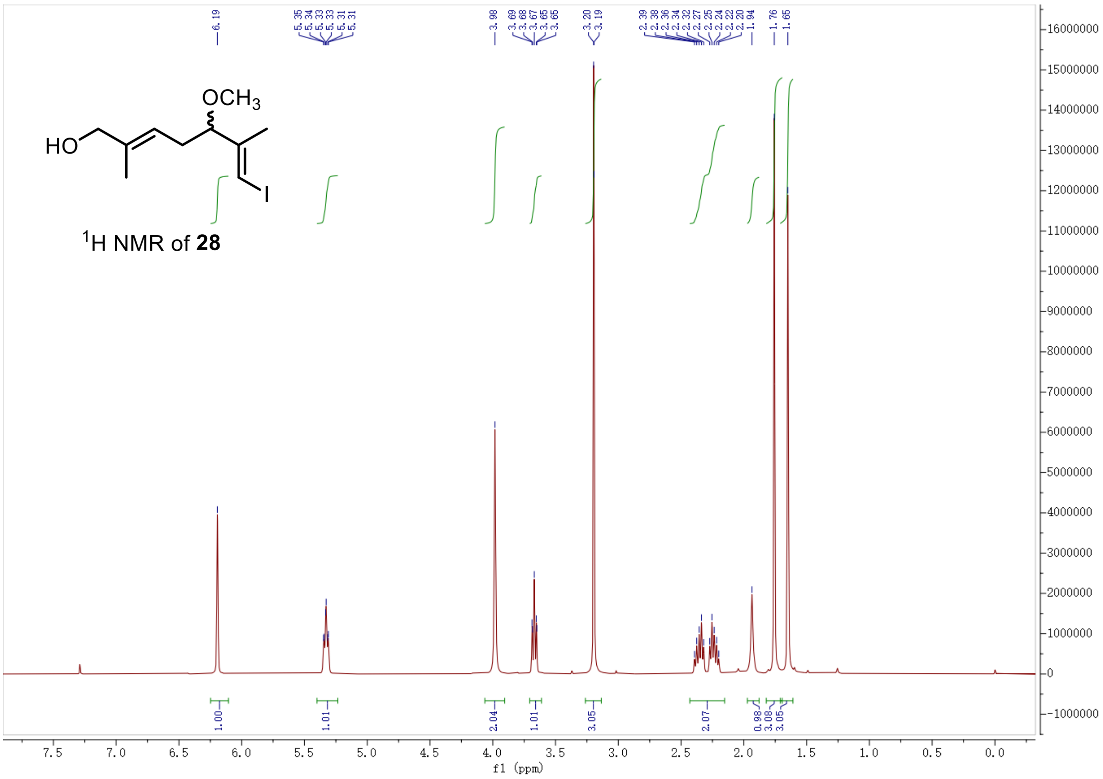
HO
OCH3
I
1H NMR of 28
f1 (ppm)

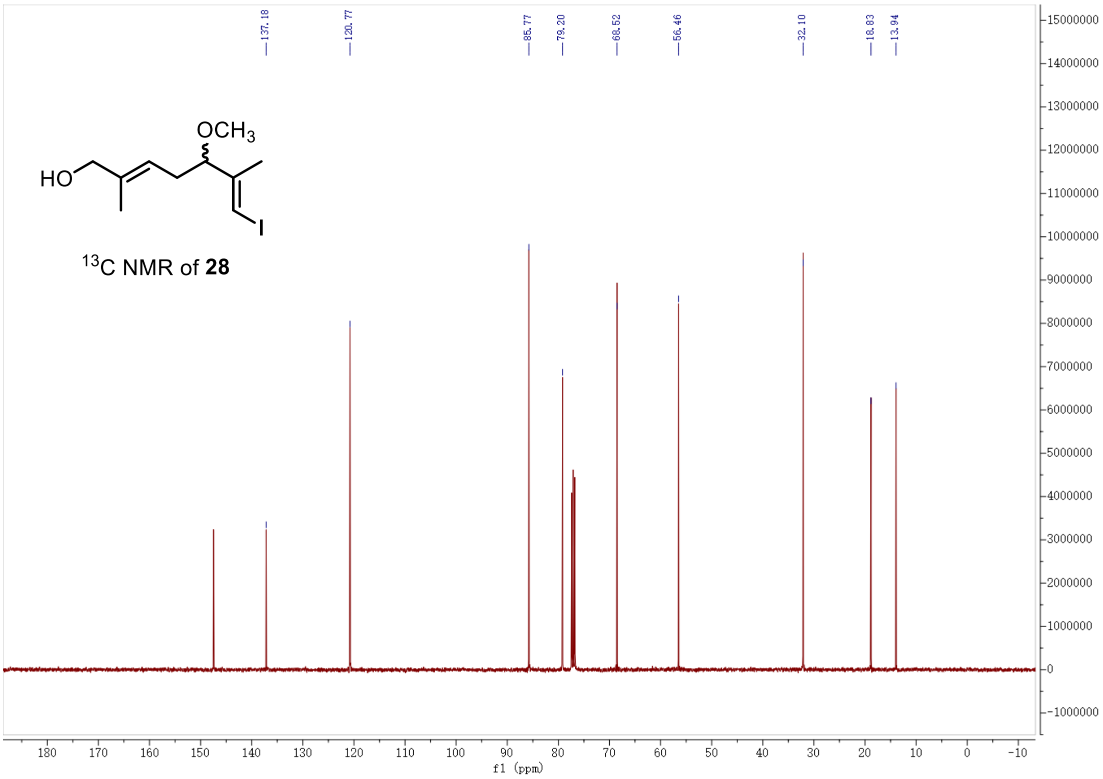
HO
OCH3
I
13C NMR of 28
f1 (ppm)

S3. The e.e. value of Mukaiyama aldol reaction is > 66% shown in ^{1}H NMR of compound 29 and chiral HPLC data of compound 26.

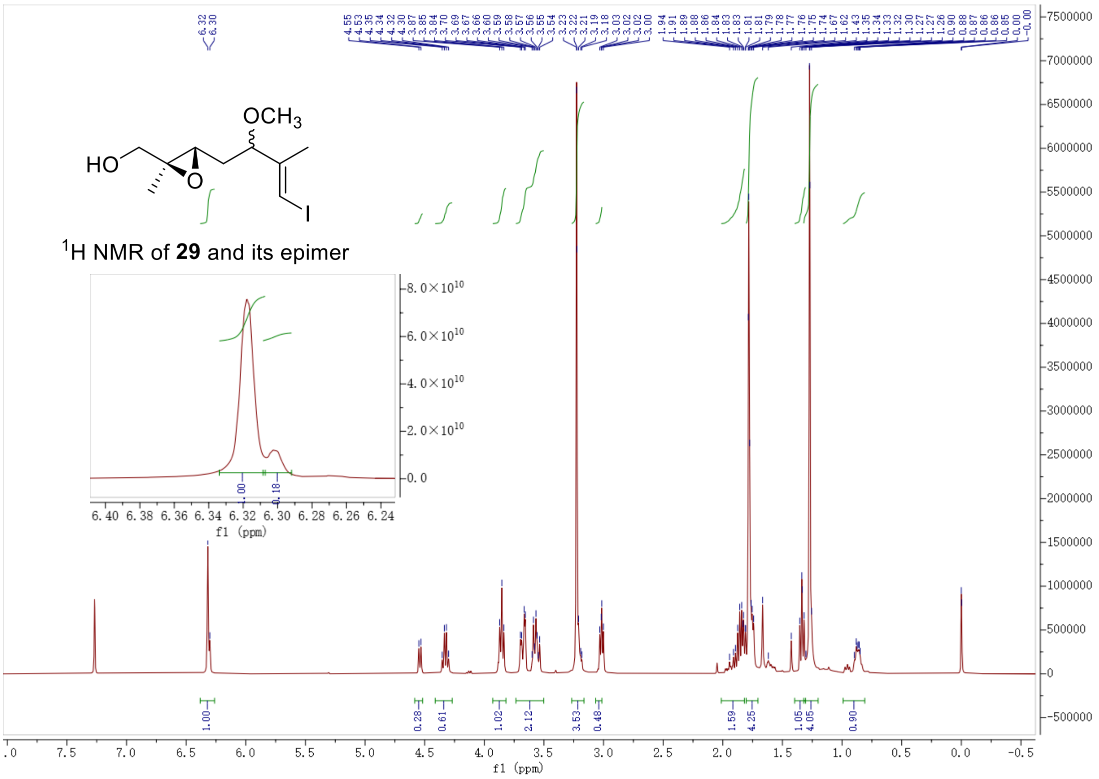

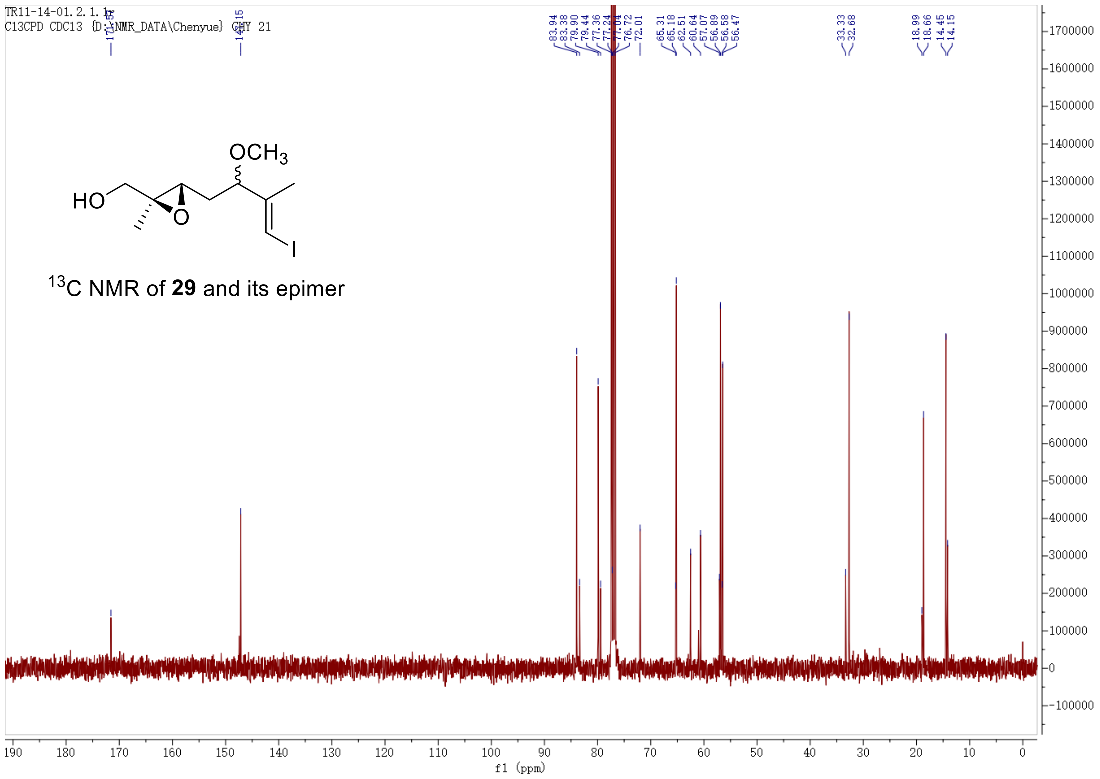

The ee value was determined by chiral HPLC (Chiralpak AD-H, 25° C, flow rate: 1 mL/min);

HPLC: 4 % isopropanol in hexane, (R)-enantiomer: retention time = 23.69 minutes, (S)-enantiomer: retention time = 18.33 minutes. The ee value of this reaction was determined as 85% (er value as 12.7:1).

HPLC for our product compound **26**:

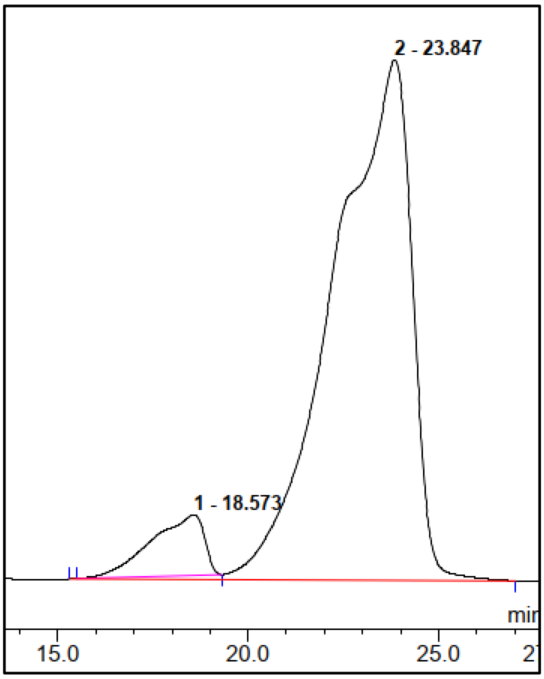

Serial Number	Retention Time	Peak Name	Peak Height /mAU	Peak Area /mAU*min	Relative Peak area/%	Sample volume	Type
1	18.57	n.a.	14.066	22.703	7.29	n.a.	Ru
2	23.85	n.a.	120.363	288.513	92.71	n.a.	BMB
Total:			134.429	311.216	100.00		

HPLC for racemates:

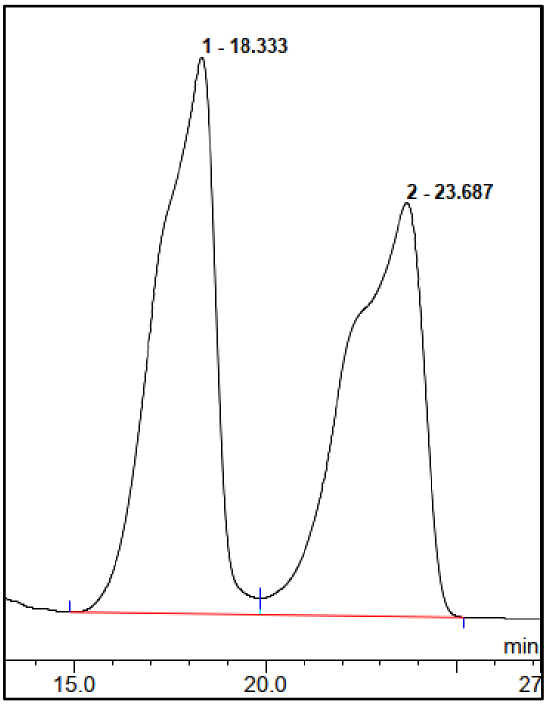

Serial Number	Retention Time	Peak Name	Peak Height /mAU	Peak Area /mAU*min	Relative Peak area/%	Sample volume	Type
1	18.33	n.a.	147.085	260.238	50.82	n.a.	BM
2	23.69	n.a.	109.367	251.866	49.18	n.a.	MB
Total:			256.452	512.105	100.00		

S4. The determination of the absolute configuration by Mosher esters of Compound 26

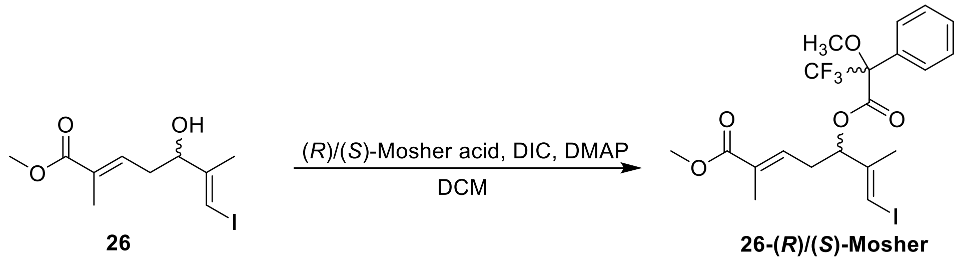

26 **26-(*R*)/(*S*)-Mosher**

To a solution of **26** (70.0 mg, 0.226 mmol, 1.2 equiv) in dry DCM (2 mL) at room temperature

was added DMAP (2.3 mg, 0.019 mmol, 0.1 equiv), (*R*) or (*S*)-Mosher acid (44 mg, 0.188 mmol, 1.0 equiv) and DIC (47 mg, 0.376 mmol, 2.0 equiv). After being stirred for 1 hour at room temperature, the reaction mixture filtered and concentrated in vacuo. The resultant was purified by silica gel column chromatography (5% EtOAc in Hexanes) to provide **26**-(*R*) and (*S*)-Mosher (89 mg, 75%) as colourless oils.

26-(*R*)-Mosher

^{1}H NMR (400 MHz, $CDCl_3$) δ 7.48 – 7.33 (m, 5H), 6.52 (d, *J* = 6.1 Hz, 2H), 5.63 (dd, *J* = 8.0, 5.7 Hz, 1H), 3.73 (d, *J* = 1.4 Hz, 3H), 3.49 (s, 3H), 2.62 (m, 1H), 2.55 – 2.42 (m, 1H), 1.85 (s, 3H), 1.74 (s, 3H).
^{13}C NMR (101 MHz, $CDCl_3$) δ 167.78, 165.66, 143.80, 134.40, 131.93, 131.07, 129.71, 128.49, 127.30, 83.23, 77.93, 55.36, 51.90, 31.99, 23.51, 20.07, 12.58.
^{19}F NMR (376 MHz, $CDCl_3$) δ -71.16.

26-(*S*)-Mosher

^{1}H NMR (400 MHz, $CDCl_3$) δ 7.42 (q, *J* = 7.3, 6.8 Hz, 6H), 6.64 (t, *J* = 7.4 Hz, 1H), 6.39 (s, 1H), 5.55 (dd, *J* = 8.7, 5.1 Hz, 1H), 3.74 (s, 3H), 3.53 (s, 3H), 2.72 – 2.58 (m, 1H), 2.58 – 2.45 (m, 1H), 1.82 (s, 3H), 1.71 (s, 3H).
^{13}C NMR (101 MHz, $CDCl_3$) δ 167.83, 165.57, 143.75, 134.85, 131.98, 131.13, 129.72, 128.47, 127.10, 82.94, 78.21, 55.65, 51.95, 32.00, 23.51, 19.89, 12.67.
^{19}F NMR (376 MHz, $CDCl_3$) δ -71.30.

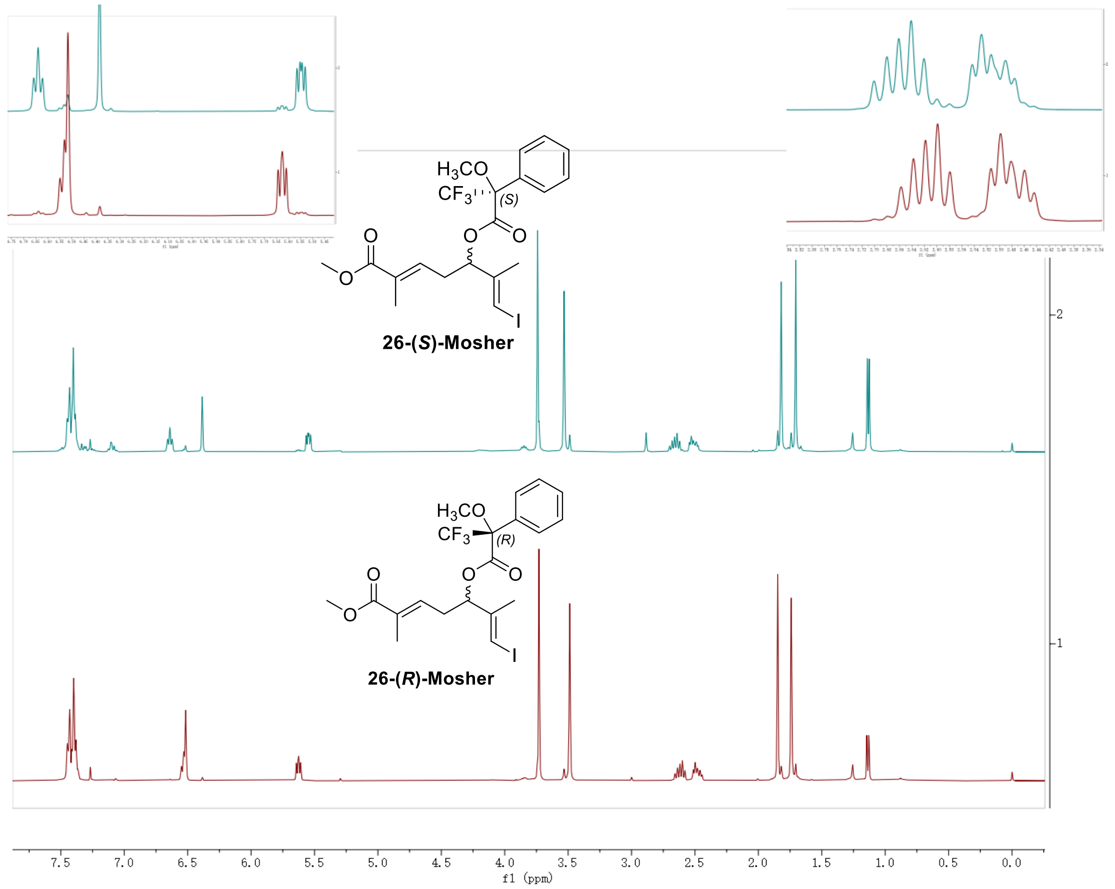

Δδ ^{SR}C4-H > 0, so the most of the absolute stereochemistry of C5 is R.

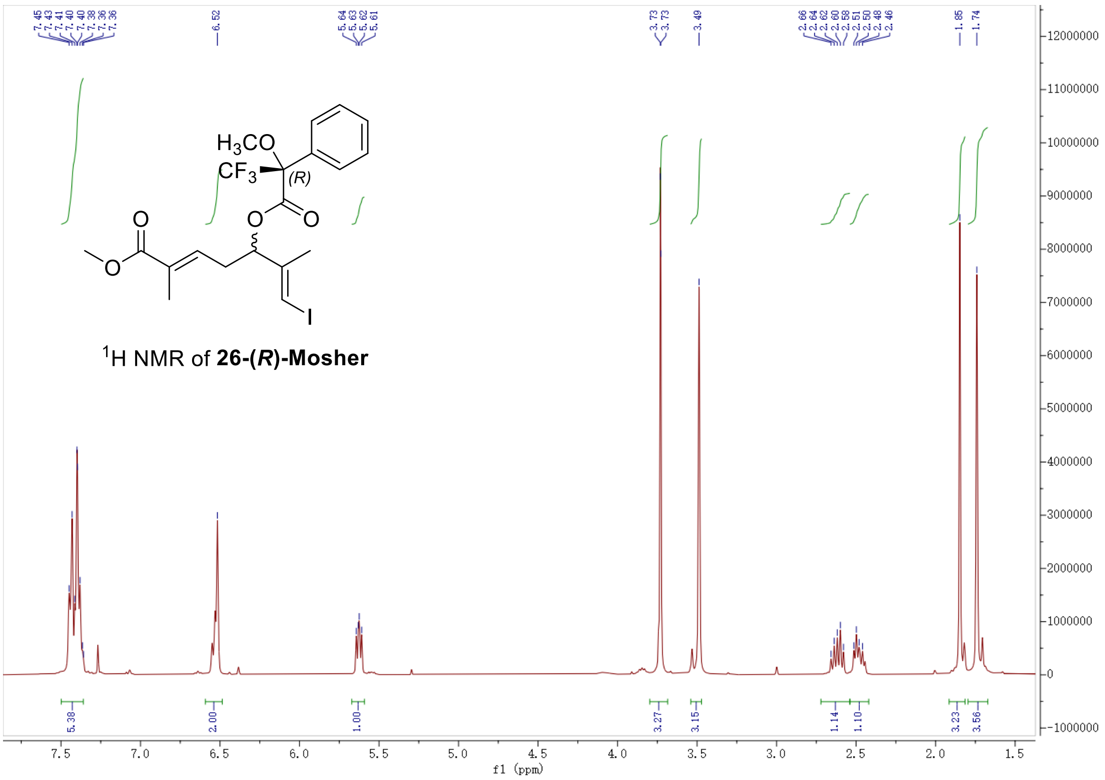

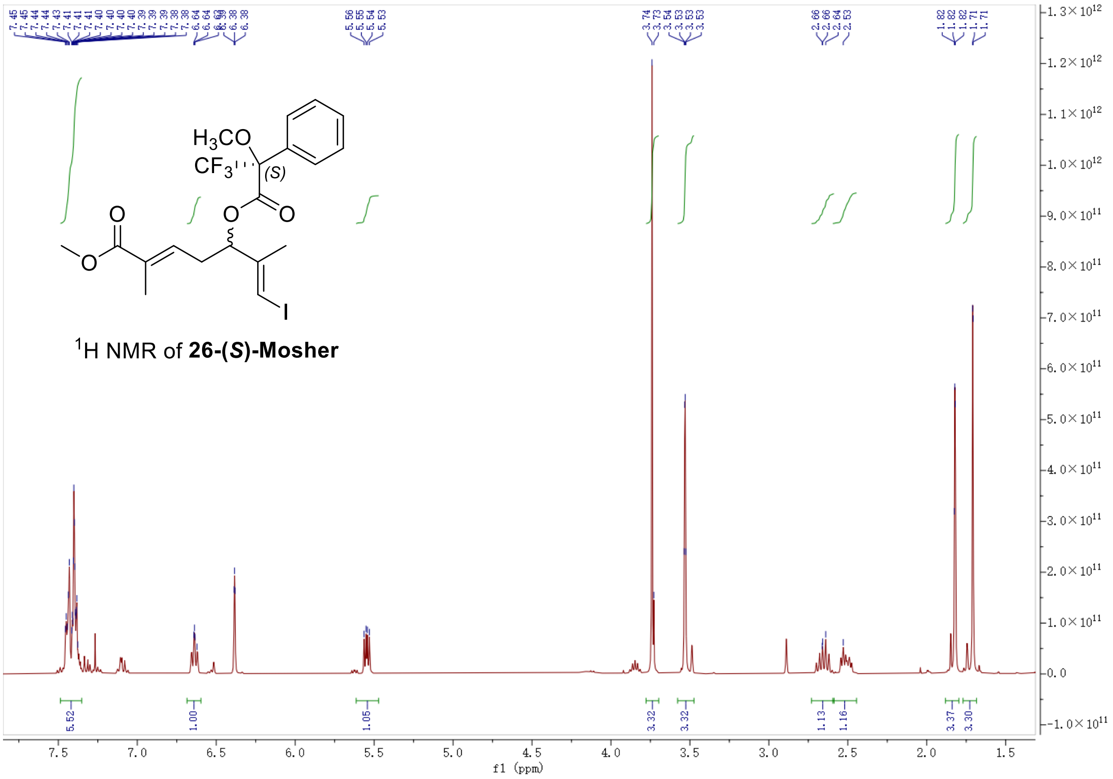

7.45 7.45 7.44 7.44 7.43 7.41 7.41 7.41 7.40 7.40 7.40 7.39 7.39 7.39 7.38 7.38 6.64 6.64 6.38 6.38
5.56 5.55 5.54 5.53
3.74 3.73 3.54 3.53 3.53 3.53
2.66 2.66 2.64 2.53
1.82 1.82 1.82 1.71 1.71
H3CO
CF3
(S)
O
O
O
O
I
1H NMR of 26-(S)-Mosher
5.52
1.00
1.05
3.32
3.32
1.13
1.16
3.37
3.30
f1 (ppm)

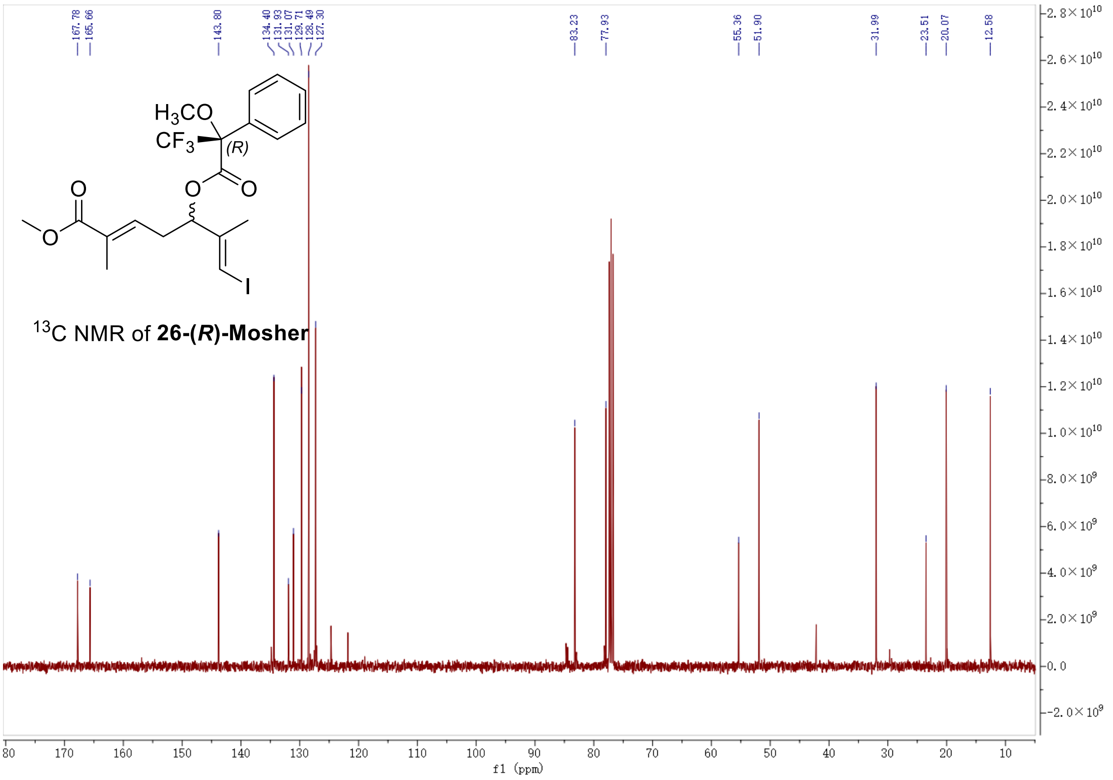

167.78 165.66
143.80
134.40 131.93 131.07 129.71 128.49 127.30
83.23
77.93
55.36
51.90
31.99
23.51
20.07
12.58
H3CO
CF3
(R)
O
O
O
O
I
13C NMR of 26-(R)-Mosher
f1 (ppm)

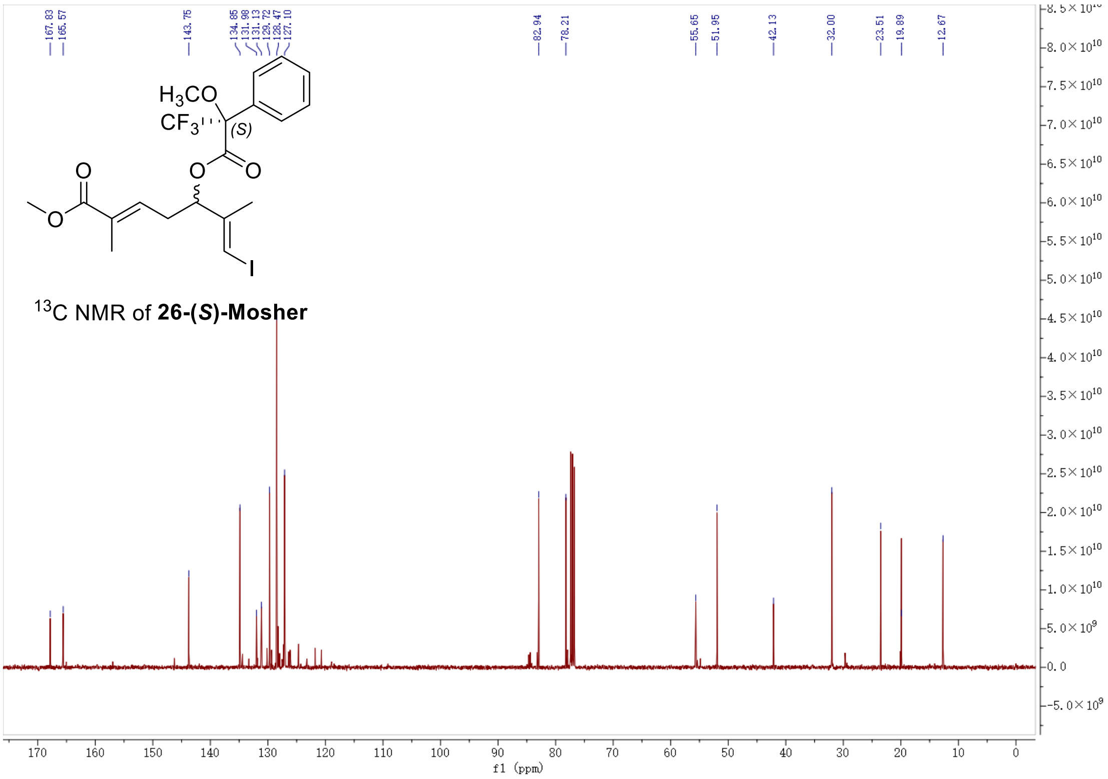
167.83
165.57
143.75
134.85
131.98
131.13
129.72
128.47
127.10
82.94
78.21
55.65
51.95
42.13
32.00
23.51
19.89
12.67
H3CO
CF3
(S)
O
O
O
I
13C NMR of 26-(S)-Mosher
f1 (ppm)

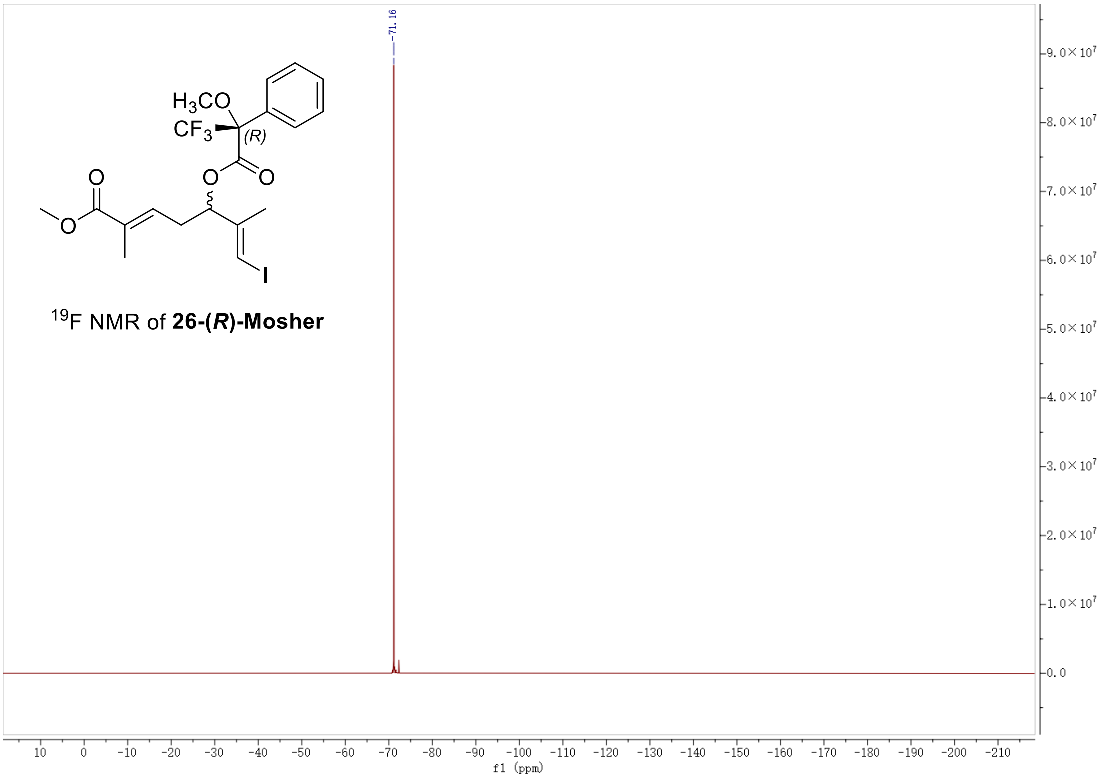
-71.16
H3CO
CF3
(R)
O
O
O
I
19F NMR of 26-(R)-Mosher
f1 (ppm)

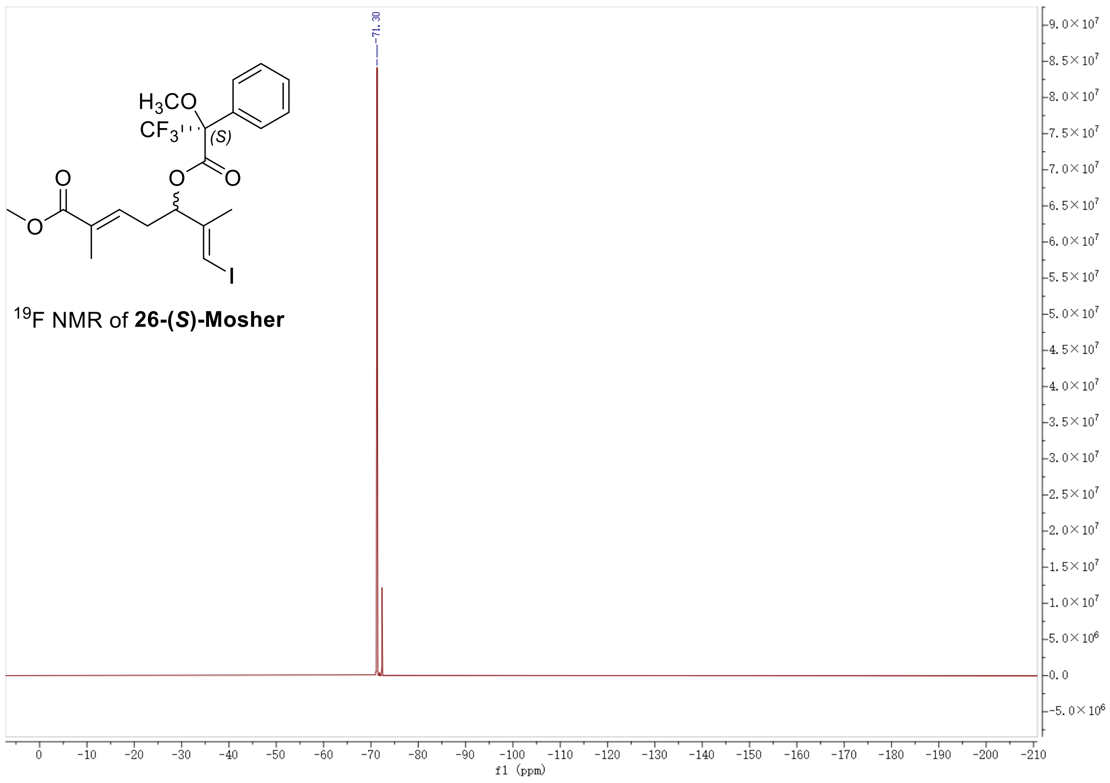

H3CO
CF3
(S)
O
O
O
O
I
19F NMR of 26-(S)-Mosher
-71.30
f1 (ppm)

S5. ¹H NMR for compound 17; 1H NMR and ¹³C NMR for nannocystin A

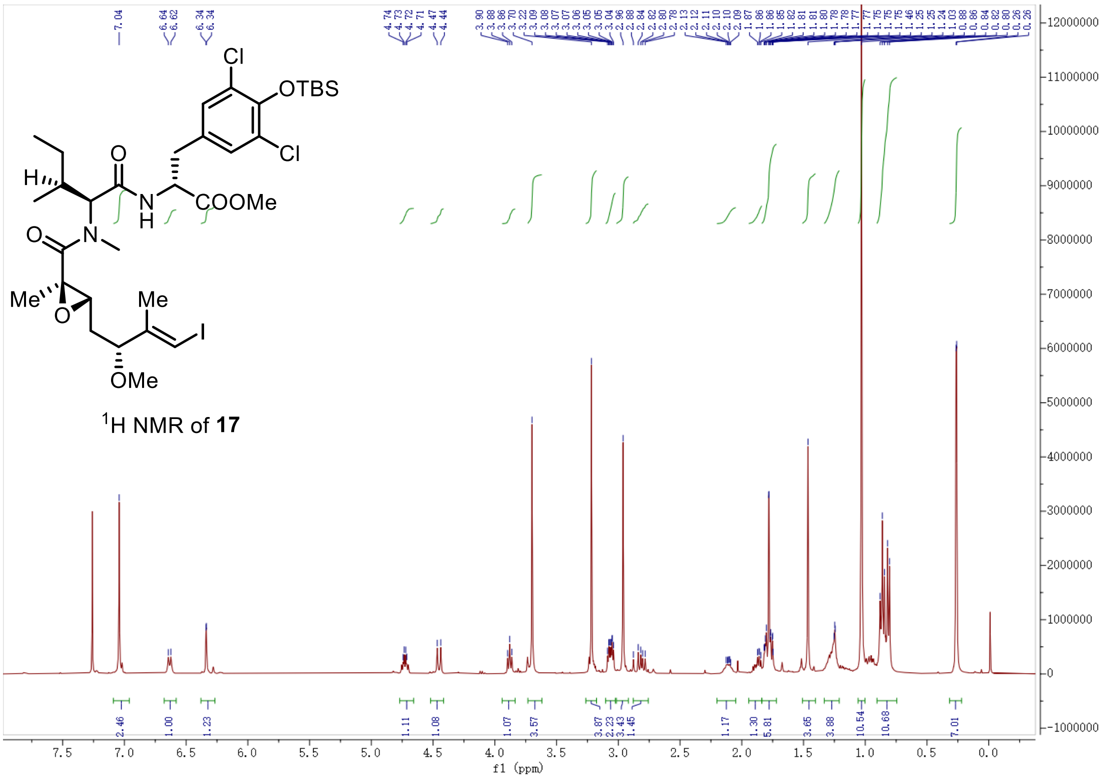

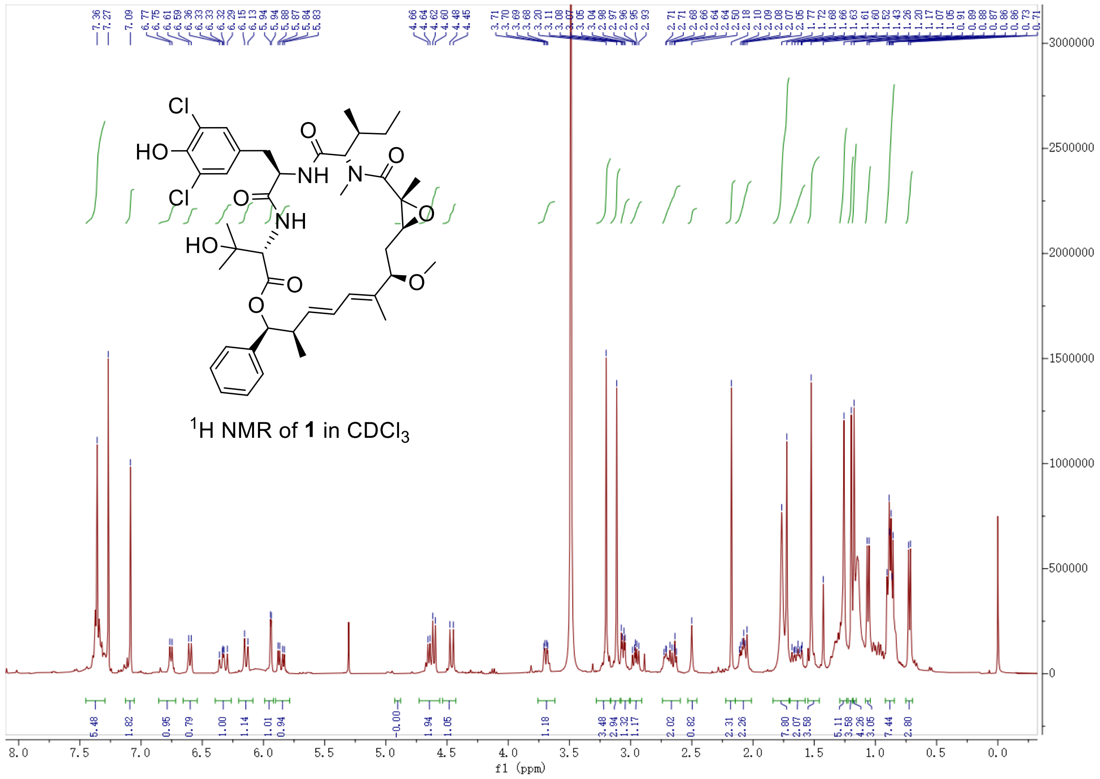
^{1}H NMR of **1** in $CDCl_3$
f1 (ppm)

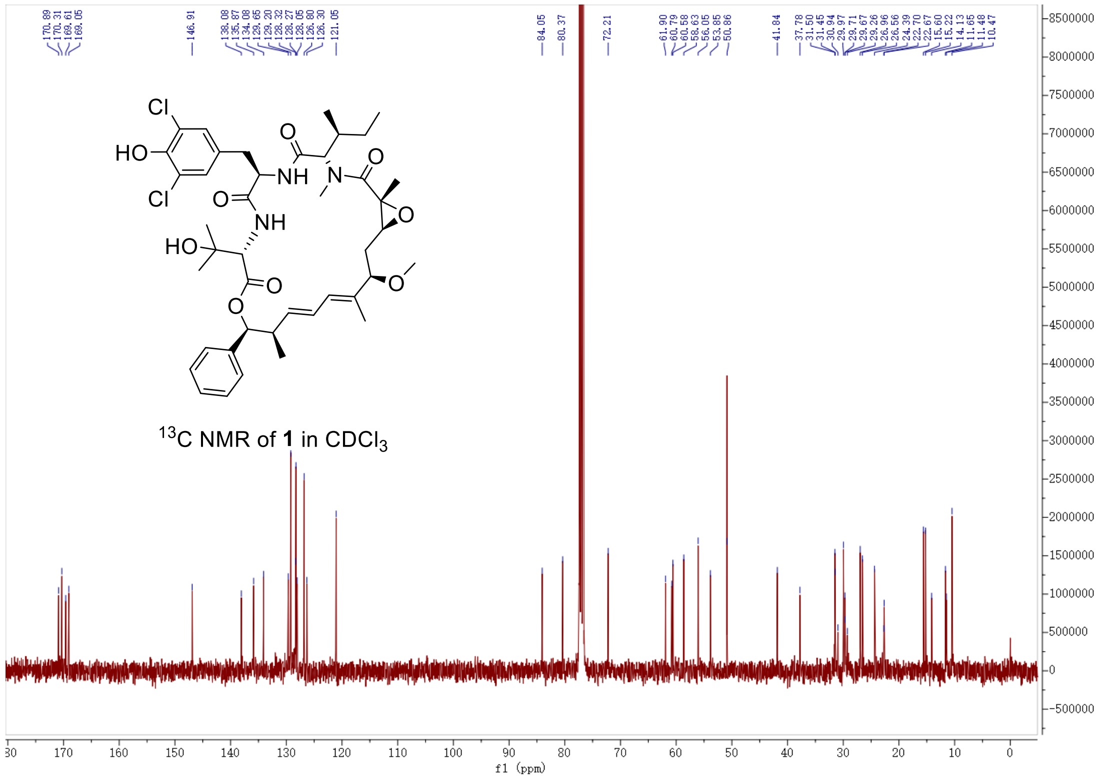
^{13}C NMR of **1** in $CDCl_3$
f1 (ppm)
